# Childbirth experience and its relationship with postpartum depression and anxiety: a cross-sectional study from Gaza

**DOI:** 10.1186/s12978-026-02291-3

**Published:** 2026-02-27

**Authors:** Alaa M. Ismail, Tayseer Afifi, Sewar A. Elejla, Mosab Samaan, Jane Elizabeth Hirst

**Affiliations:** 1 Faculty of Medicine, Al Quds University, Gaza, Palestine; 2 Faculty of Medicine, Islamic University, Gaza, Palestine; 3 Faculty of Health and Medicine, Department of Psychiatry, McGill University, Montreal, Canada; 4 Nuffield Department of Women’s & Reproductive Health, University of Oxford, Oxford, UK; 5 The George Institute for Global Health, Imperial College, London, UK

**Keywords:** Childbirth experience, Obstetric maltreatment, Postpartum depression and anxiety, Gaza, Palestine

## Abstract

**Background:**

The quality of childbirth experience has a profound effect on maternal mental health. Negative experiences during labour, including poor communication, lack of informed consent, and obstetric maltreatment, can increase the risk of postpartum depression (PPD) and postpartum anxiety (PPA). This study explores women’s perspectives on the childbirth experience in Gaza and its associations with PPD and PPA.

**Aims:**

To explore women’s perspectives on the childbirth experience and its relationship with the development of postpartum depression and anxiety. The study included women who gave birth at Gaza hospitals between 2017 and 2021.

**Methods:**

This cross-sectional study surveyed 722 women who gave birth between January 2017 and December 2021 in Gaza’s public and private hospitals. Data were collected via a self-administered online questionnaire shared via social media and working groups. The tool included five sections: sociodemographics, childbirth experience (pain score, satisfaction, communication, procedures maltreatment practices, and postpartum mental health, which were assessed via the PHQ-2 and GAD-2 screening tools).

**Results:**

The mean participant age was 27.7 years (SD ± 5.37). Most births occurred in governmental hospitals (70.3%), with normal vaginal delivery being most common (74.4%). While 87.1% had regular antenatal care, 49% reported inadequate information on labour, and 29.6% received no explanation regarding vaginal exams. A majority (68.6%) were unaware of episiotomy before the procedure, and 66.9% underwent unconsented episiotomies, often without local anaesthesia (30.6%). Obstetric maltreatment was an unfamiliar term for 41.5% of the women. Inadequate analgesia was the most reported form (63.4%). Compared with those not exposed (*n* = 422, 58.4%) (*p* = 0.001), women exposed to any form of maltreatment (*n* = 300, 41.5%) were at increased risk for PPD or PPA. Among those who were exposed to maltreatment, 78.7% (*n* = 236) reported PPD and PPA. Instrumental vaginal delivery (AOR = 3.25; *p* = 0.00, 95% CI = 2.04–4.24) and elective caesarean section (AOR = 2.01; *p* = 0.001, 95% CI = 1.84–5.17) significantly increased the risk of PPD. Similarly, PPA was more prevalent among women who had elective CS (AOR = 9.16; *p* = 0.001, 95% CI = 6.32–17.24) and IVDs (AOR = 3.71; *p* = 0.001, 95% CI = 2.17–5.68). Women who reported being aware of obstetric maltreatment were less likely to develop PPD (AOR = 0.46; *p* = 0.04, 95% CI = 0.21–0.87) and PPA (AOR = 0.36; *p* = 0.01, 95% CI = 0.17–0.76).

**Conclusion:**

PPD and PPA were associated with negative childbirth experiences, particularly maltreatment and lack of informed care. Promoting respectful maternity care and improving patient education may reduce these mental health risks.

## Background

Pregnancy and childbirth are challenging events. Childbirth brings a combination of psychological, emotional, physical and social events that affect not only the woman and her baby but also the entire family. A stressful or traumatic childbirth experience can have short- and long-term implications for future reproductive outcomes, such as breastfeeding, postpartum recovery and subsequent deliveries [1] Click or tap here to enter text.. Factors such as pain, communication and counselling, the presence of companions, receiving adequate analgesia, perceived control, sharing decisions with caregivers, mode of delivery and many others contribute significantly to postpartum recovery and longer-term maternal wellbeing [2–5].

Respectful maternity care should be a universal right for all women in every maternity setting. Respectful care starts prenatally and continues through labour, birth and postnatally. The treatment of women has emerged as a global public health problem with a relatively high prevalence in low- and middle-income countries (LMICs). The WHO has stated that maltreatment during childbirth is a violation of rights and trust between women and their caregivers. In one study, 35% of 2672 women across four countries were exposed to at least one form of verbal, physical or psychological abuse [6, 7]. We previously reported a high rate of obstetric violence in Gaza and major predisposing risk factors [8].

Negative childbirth experiences, including feeling dismissed and neglected, birth difficulties, traumatic obstetric procedures, loss of support, and loss of pain control, can predispose individuals to depression and anxiety, breastfeeding difficulties, and disruption in sexual activity [9, 10]. Postpartum depression (PPD) is considered a leading cause of disability globally, with an estimated prevalence of 20% in low- and middle-income countries [7]. It comprises a spectrum of symptoms ranging from loss of interest, loss of concentration and productivity, insomnia and difficulty in bonding with the baby to suicidal thoughts and harm to oneself and the baby in its severest form. Furthermore, postpartum anxiety (PPA) includes intrusive worries and fears that hinder normal daily activities, including caring for oneself and the baby. In one meta-analysis, the prevalence of PPA ranged between 4% in low-risk women and 18.9% in high-risk women [11].

Gaza is a highly condensed area with a crude birth rate of 32 births/1000 and nearly 57,877 live births recorded in 2022 [12, 13]. The rates of mental health problems during pregnancy and childbirth in Palestine have been increasing in recent years, ranging from 12 to 36%, with higher rates in Gaza than in the West Bank due to severe social restrictions and the political situation [14–16]. The factors contributing to the higher rate of PPD in Gaza included the stress of living in a conflict setting, low social class and poverty, unplanned pregnancy, and maltreatment during childbirth [17].

Since October 2023, the people of Gaza have endured one of the most devastating wars in recent history. Forced displacements, social unrest, high casualty figures, and ongoing hostilities have caused the healthcare system to collapse. Women, who are especially vulnerable during pregnancy and childbirth, have been disproportionately affected. Many have been compelled to give birth in tents and on the streets or to labour without skilled attendants. Most deliveries have taken place under difficult conditions, lacking essential resources and medicines. The lack of adequate antenatal and postnatal care, combined with ongoing conflict and violence, has heightened women's risk of preventable health issues and postpartum psychological problems.

Although the optimal standards for childbirth care have been defined by the World Health Organization (WHO) [18], the realistic application of evidence-based practices and local policy is highly challenging in conflict-affected and resource-poor regions such as Gaza. We have previously described the types of maltreatment reported by women during pregnancy and childbirth in Gaza before the current war [19]. In this study, we aimed to determine the associations between different forms of maltreatment during pregnancy and delivery care and the development of postpartum depression and anxiety. We describe women’s perceptions of the childbirth experience in Gaza hospitals, the obstacles women face in seeking care, and their perspectives on common practices and procedures performed during delivery.

## Methods

### Study design

This was a cross-sectional study. An online, self-administered questionnaire was used to collect data on childbirth experiences, common practices during delivery and the relationship between women’s experience and the risk of postpartum depression and anxiety.

### Setting

This study was conducted in Gaza from November 2021 to February 2022.

### Participants

We actively recruited women who delivered during the preceding 5 years from January 2017 to December 2021 and encouraged them to self-complete the questionnaire after providing consent. Inclusion criteria of participants included: 1. Being a resident in Gaza; 2. Has delivered in the preceding five years from the time of data collection; 3. Women of all ages including teenagers and old women; 4. All delivery settings including governmental, private and home births; 5. Ability to reach out the online questionnaire and self-administer it.

### Variables

Our primary outcome of interest was the risk of mental health disorders recalled by women in the first few weeks after giving birth. Depression and anxiety were assessed via the Patient Health Questionnaire (PHQ-2) and the Generalized Anxiety Disorder Scale (GAD-2) as brief screening measures instead of the 9-point scale, which has similar sensitivity and specificity [20–22]. Each score has two screening questions; each is scored from 0 to 3. The total score ranges from 0 to 6. A score of three or more is used as a cut-off point for potential depression or anxiety. A positive screening test necessitates thorough evaluation and management.

The key exposure of interest for this study was self-reports of maltreatment during pregnancy and birth. Women were asked if they experienced any of the seven most common forms of disrespectful care during their most recent pregnancy and birth: discrimination, nonconsent procedures, breach of confidentiality and privacy, negligence, disrespect and inadequate medical care, not allowing companions throughout the labour journey, or inadequate pain control [23]. Women were considered to have experienced maltreatment if they answered yes to any of these experiences.

We captured potential other predictors, potential confounders and effect modifiers of the relationship between maltreatment during pregnancy and birth and mental health through the questionnaire, including questions on sociodemographic characteristics, mode and outcome of birth, intrapartum care procedures, knowledge of caregivers, pain perception, and postnatal recovery.

To minimize the potential effects of recall bias on mental health, we conducted a subgroup analysis of women who delivered within 12 months of completing the survey.

### Data sources

All the data were captured through an online survey (Google form), and the results were stored in Microsoft Excel. The data were self-reported, and no verification of the answers was performed.

Whilst women completed the form independently, data collectors were available online to provide explanations and ensure understanding of questions.

### Study size/precision

This analysis was a planned exploratory analysis of the survey data. Prior work indicated an exposure prevalence (obstetric violence) of 33.3% and an expected prevalence of postpartum depression and/or anxiety (PPD/PPA) of 25%. Given the sample size of 722, we calculated precision for two complementary goals: (1) precise estimation of the overall prevalence of PPD/PPA, and (2) adequate power to detect differences in PPD/PPA between women exposed versus not exposed to obstetric maltreatment.

1. Precision for prevalence: for a single-proportion 95% CI, we used the formula n = Z^2^ × p(1-p)/d^2^, where Z = 1.96, p is the anticipated prevalence, and d is the desired half-width (margin of error). Using *p* = 0.25, for a margin d = 0.037 (± 3.7%) 526 participants would be required. Thus, our sample of 722 gives a 95% CI half-width for *p* = 0.25 of approximately ± 3.16%.
2. Power for comparing exposed vs unexposed (binary outcome). For a two-group comparison with unequal group sizes (exposure prevalence ≈33.3%), we used a normal-approximation formula. As a representative effect size we considered an odds ratio ≈2.0 (corresponding to an increase from 25% in the unexposed to 40% in the exposed group; absolute difference 15%). With α = 0.05 and 80% power, the required total sample is ≈350 (exposed ≈116; unexposed ≈233). For a smaller absolute difference of 10% (25% → 35%), the required total sample is ≈757.

Accordingly, a conservative target of ~ 700 participants was chosen; the final analysed sample of 722 provides good precision for prevalence estimation (95% CI half-width ± 3.2%) and adequate power to detect moderate or larger differences (≈15% absolute difference, OR≈2) between exposure groups, though it would be underpowered to detect smaller absolute differences (≈10%) with 80% power.

### Quantitative variables

Continuous variables are presented as the means and standard deviations, whereas categorical variables are presented as frequencies and percentages.

### Statistical methods

The data were entered into SPSS version 23 and then cleaned prior to analysis. Chi square tests were used to assess relationships between categorical variables (independent variables with exposure to maltreatment as well as depression and anxiety). Multiple logistic regression was performed to determine the associations between the dependent and independent variables. A *P* value less than 0.05 was considered significant. As this was an exploratory analysis, there were no adjustments in significance levels made for multiple testing. There was no imputation performed for missing variables.

## Results

### Participants’ sociodemographic and obstetric characteristics

The study included 722 participants. The majority of the participants were aged 21–30 years (*n* = 483, 67.2%) and were married (*n* = 716, 99.2%). Most were unemployed or housewives (*n* = 532, 73.7%), with husbands primarily employed (*n* = 433, 60%). Gaza city was the most common place of residence (*n* = 313, 43.4%), and most lived in urban areas (*n* = 513, 71.1%). Half had a monthly income less than 1000 shekels (*n* = 362, 50.1%). Among the multiparous women, most had 1–3 previous deliveries (*n* = 387, 81.6%). The majority of women had delivered in government hospitals (*n* = 508, 70.4%) via normal vaginal delivery (*n* = 537, 74.4%). Most women had no prior knowledge of their attending labour staff (*n* = 505, 69.9%); however, they did know whether an obstetrician or midwife was present at birth (*n* = 442, 61.2%). Most women had regular antenatal follow-up during pregnancy (*n* = 629, 87.1%); however, only half reported that they had received information about the potential for labour difficulties (*n* = 354, 49%). Self-education about labour during pregnancy was reported by 69.9% (*n* = 505) of the participants. Table 1 summarises the sociodemographic and birth-related characteristics of the study participants.

**Table 1:** Demographic characteristics of participants. This table summarizes the demographic profile of participants, including age, marital status, employment, and obstetric history

Variable	N (%)/Mean ± SD
Age (Mean ± SD, min–max)	27.7 ± 5.37 (14–47)
Under 20	29 (4.0%)
21–30	483 (67.2%)
31–40	192 (26.7%)
41–50	15 (2.1%)
Married	716 (99.2%)
Divorced	5 (0.7%)
Widowed	1 (0.1%)
Employed	125 (17.3%)
Unemployed/Housewife	532 (73.7%)
Part-time Job	63 (8.7%)
Retired	2 (0.3%)
Husband Employed	433 (60.0%)
Husband Unemployed	99 (13.7%)
Husband Part-time	180 (24.9%)
Husband Retired	10 (1.4%)
Urban	513 (71.1%)
Camp	209 (28.9%)
< 1000 shekel	362 (50.1%)
2000–2900 shekel	73 (10.1%)
> 3000 shekel	54 (7.5%)
1–3 Deliveries	387 (81.6%)
4–6 Deliveries	75 (15.8%)
6–9 Deliveries	8 (1.7%)
> 9 Deliveries	2 (0.4%)
Governmental Hospital	508 (70.4%)
Private Medical Center	33 (3.0%)
Private Hospital	188 (26.0%)
Home	4 (0.6%)
Normal Delivery	537 (74.4%)
Elective Cesarean	75 (10.4%)
Emergency Cesarean	79 (10.9%)
Ventose/Forceps	31 (4.3%)
Regular OB Follow-up	629 (87.1%)
Explained Labor Difficulties	354 (49.0%)
Self Education During Pregnancy	505 (69.9%)

### Childbirth experience and procedural characteristics

Pain expectations were assessed via a numeric rating scale (0–10). Prior to labour, 73.3% (*n* = 529) anticipated severe pain (score ≥ 7), whereas after delivery, this figure rose to 91.3% (*n* = 659).Regarding vaginal examination (PV), 85.6% (*n* = 618) of the patients underwent PV at some point during pregnancy or labor. However, 29.6% (*n* = 214) stated that they received no information about their purpose or process. Consent was obtained from only 67.4% (*n* = 487), and 9.5% (*n* = 69) reported that PV was performed without ensuring full privacy.

More than half of the women (*n* = 385, 53.3%) had no prior knowledge of episiotomy, and 68.6% (*n* = 495) reported that the procedure was not explained during antenatal visits. A significant proportion of episiotomies (66.9%, *n* = 483) were performed in the presence of trainees without consent, and 30.6% (*n* = 221) reported no use of local anaesthesia during repair. Additionally, only 30.3% (*n* = 219) were provided with a delivery gown, with most giving birth in their personal clothing Table 2.

**Table 2:** Women’s perspectives on common obstetric procedures during birth

Statement	Response (N, %)
Knowledge about vaginal examination	Yes 391 (54.2%)/No 259 (35.9%)/Maybe 72 (10.0%)
Importance explained by obstetrician	Yes 214 (29.6%)/No 465 (64.4%)/Maybe 43 (6.0%)
Consent before vaginal examination	Yes 421 (58.3%)/No 131 (18.1%)/Sometimes 66 (9.1%)
Privacy during examination	Yes 472 (65.3%)/No 69 (9.5%)/To some extent 77 (10.6%)
Knowledge of episiotomy	Yes 292 (40.4%)/No 385 (53.3%)/Maybe 45 (6.2%)
Episiotomy explained by obstetrician	Yes 187 (25.9%)/No 495 (68.6%)/Maybe 40 (5.5%)
Use of anaesthesia in episiotomy	Yes 341 (47.2%)/No 221 (30.6%)/Don’t know 160 (22.2%)
Consent for trainee attendance	Yes 166 (23.0%)/No 483 (66.9%)/Sometimes 73 (10.1%)
Labor clothing for privacy	Yes 219 (30.3%)/No 472 (65.4%)/Don’t know 31 (4.3%)
Awareness of labour clothing	Yes 309 (42.8%)/No 367 (50.8%)/Maybe 46 (6.4%)

### Experience of obstetric maltreatment

**Fig. 1:**
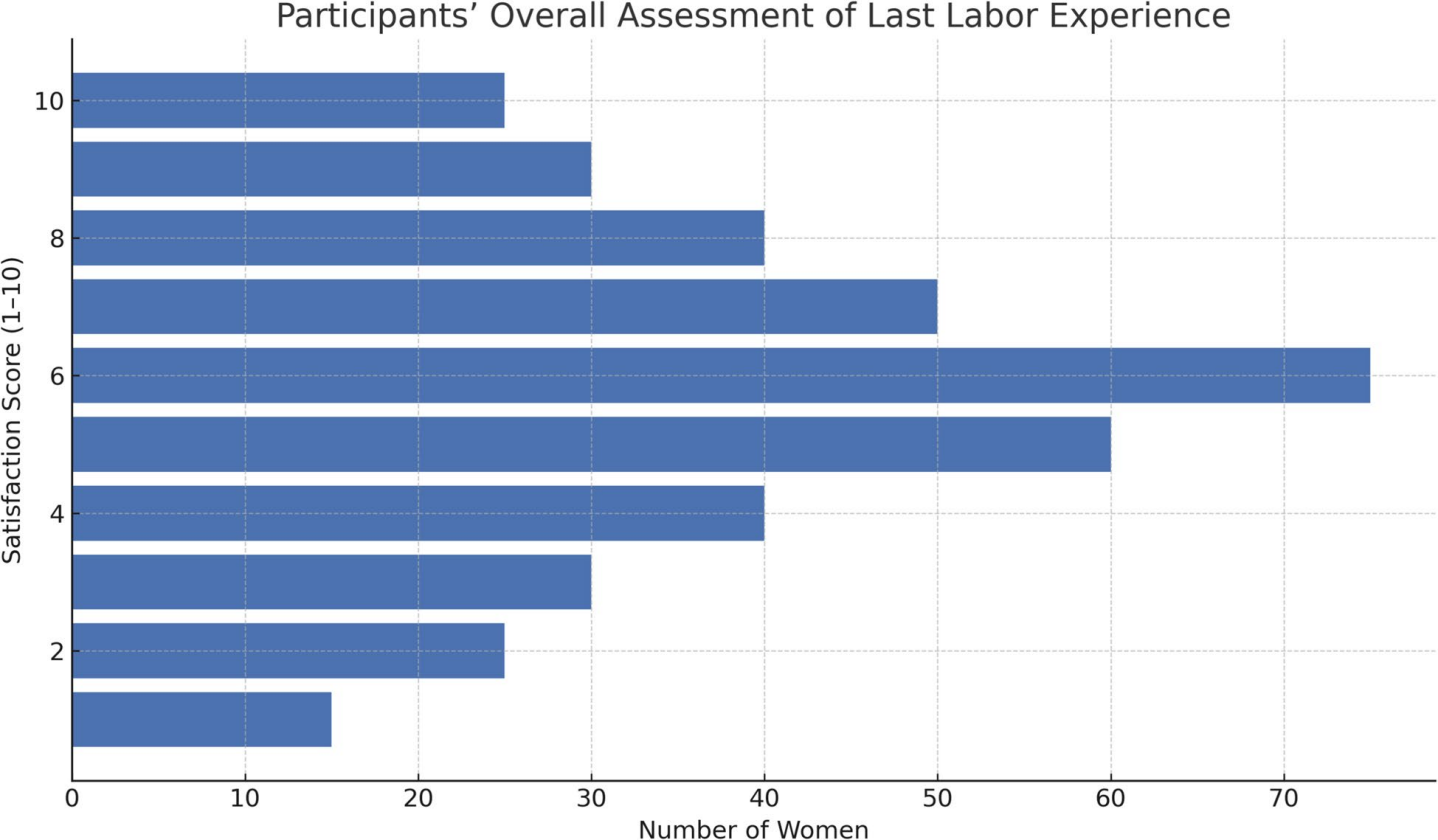
Participants’ overall assessment of their last labour experience

After explaining the forms and definitions of obstetric maltreatment to women, 41.5% (*n* = 300) reported experiencing at least one form of obstetric maltreatment. The most common issues included inadequate pain relief (*n* = 458, 63.4%), the absence of a companion during labour (63%), a lack of staff availability (48.7%), poor communication and counselling (41.8%), and insufficient resources (38.5%). Other reported problems were unconsented procedures (36.7%), compromised privacy (35.3%), and exposure to multiple trainees without adequate privacy (33.3%). Despite these challenges, 60.6% of women (*n* = 438) rated their overall experience as positive (score ≥ 5 out of 10) Fig. 1.

Women unaware of the identity of their caregiver during delivery were significantly more likely to report maltreatment (48.7% vs. 21.9%, *p* < 0.001). Similarly, women who did not know whether an obstetrician or a midwife attended their delivery were significantly more likely to report maltreatment (48.7% vs. 21.9%, *p* < 0.001) Table 3.

**Table 3:** Common forms of obstetric maltreatment

Statement	N (%)
Ever heard of 'Obstetric maltreatment'	Yes 346 (47.9%)/No 300 (41.5%)/Maybe 78 (11.6%)
Analgesics not enough	458 (63.4%)
No companion allowed	455 (63.0%)
Staff unavailable when needed	352 (48.7%)
Insufficient delivery explanation	302 (41.8%)
Lack of hospital resources	278 (38.5%)
No consent during procedures	265 (36.7%)
Privacy not considered	255 (35.3%)
Many trainees present	241 (33.3%)
Other	16 (2.2%)

### Postpartum depression (PPD) and postpartum anxiety (PPA)

**Fig. 2:**
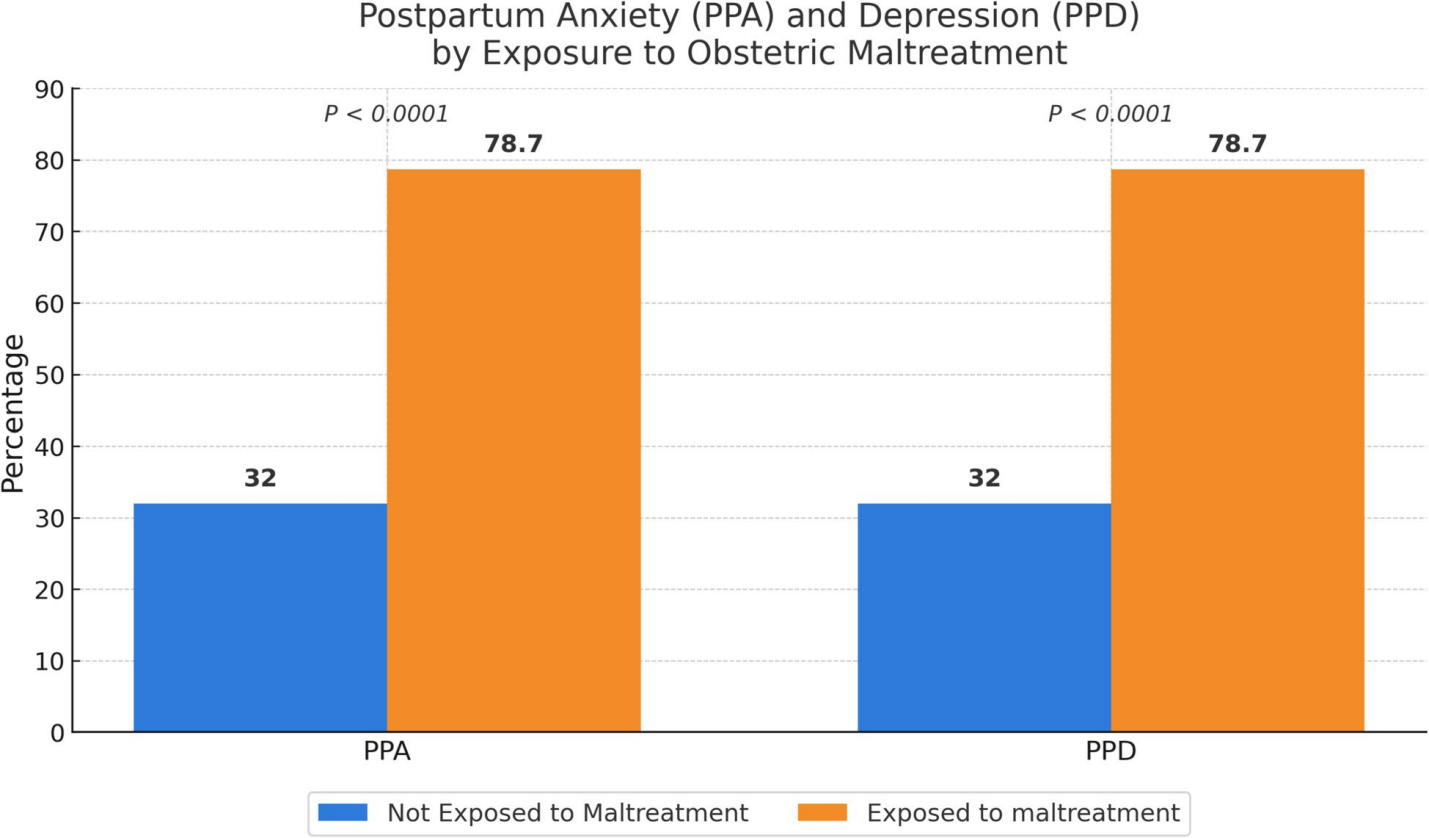
Percentage of PPA and PPD among women based on exposure to maltreatment

A total of 510 participants reported above the threshold risk for PPA (70.6%), and 511 women reported above the threshold risk for PPD (70.8%). Among women who were exposed to obstetric maltreatment (*N* = 300, 41.6%), 78.7% (*n* = 236) scored above the threshold for the risk of PPD and PPA (*p* < 0.0001) (Fig. 2). The number of women not exposed to maltreatment was 422 (58.4%).

Univariate analysis revealed several risk factors for PPD and PPA: low socioeconomic status, multiparity (1–3 previous deliveries), vaginal delivery, and prior knowledge of labour difficulties. Severe labour pain (score of ≥ 7) was associated with a greater risk of PPA.

Multivariate analysis revealed that women who underwent IVDs (AOR = 6.62; CI: 1.47–26.56; *p* = 0.013) were more likely to develop PPD. Elective CS (AOR = 9.16; CI: 2.66–10.14; *p* = 0.001) and IVDs (AOR = 3.71; CI: 2.71–5.71; *p* = 0.001) were also strong predictors of PPA. Conversely, awareness and education about obstetric maltreatment were protective, reducing the risk of PPD (AOR = 0.46; CI: 0.21–1.00; *p* = 0.04) and PPA (AOR = 0.36; CI: 0.16–0.78; *p* = 0.01).

Within the subgroup of women who delivered within 12 months prior to data collection, similar associations were observed. Women who underwent elective caesarean sections had significantly greater odds of postpartum depression (AOR = 5.65, 95% CI: 1.192–6.86). Additionally, women with high parity (6–9 previous deliveries) were also more likely to experience depression (AOR = 3.139, 95% CI: 2.147–8.254). Notably, awareness of labour maltreatment was significantly associated with decreased odds of postpartum anxiety (AOR = 0.94, 95% CI: 0.84–1.04, *P* = 0.04). Other variables, including age, socioeconomic status, and pain perception, were not significantly associated with depression or anxiety in this subgroup Table 4.

**Table 4:** Sub-group analysis for women delivering within 12 months prior to data collection (*N* = 290). Multivariable logistic regression analysis showing adjusted odds ratios (AOR) and 95% confidence intervals (CI) for predictors of depression and anxiety

Variable	Depression (AOR)	95% CI	Anxiety (AOR)	95% CI
21–30	1.20	0.95–3.54	1.82	0.06–5.19
31–40	0.91	0.08–9.61	1.70	0.18–2.64
41–50	1.45	0.15–14.04	1.64	0.57–1.02
4–6 parity	0.61	0.20–1.79	0.60	0.20–1.84
6–9 parity	3.13	2.14–8.25	1.36	0.07–2.38
> 9 parity	0.11	0.02–0.59	0.75	0.34–1.24
SES 1000–1900	0.45	0.12–1.68	0.60	0.16–2.23
SES ≥ 2000	2.04	0.82–5.07	1.55	0.63–3.81
Urgent CS	1.25	0.30–5.09	2.42	0.68–8.64
Elective CS	5.65	1.19–6.86	0.29	0.06–1.32
Assisted Vaginal	0.56	0.21–1.65	0.63	0.14–3.25
Read/Watch labor docs	0.91	0.73–1.96	0.92	0.48–1.08
Awareness of maltreatment	0.92	0.40–2.15	0.94	0.84–1.04
Pain before labor	0.90	0.30–2.72	1.01	0.35–2.86
Pain after labor	0.23	0.03–1.73	0.71	0.13–3.86
Maltreatment exposure	1.54	0.65–3.63	0.84	0.35–1.99

## Discussion

We highlight the importance of perceived childbirth experiences with subsequent reports of symptoms of postnatal depression and/or anxiety. We show that both the prevalence and impact of obstetric maltreatment are common in Gaza and identify key sociodemographic and obstetric factors that contribute to or protect against PPA and PPD. Although the link between maltreatment and postpartum mental health outcomes is well-established, few studies have specifically examined how women in Gaza are impacted. We highlight that respectful maternity care must remain central to obstetric services even in conflict zones, where women face ongoing security and existential threats to their lives and livelihoods. Understanding the specific types of maltreatment women experience that are linked to PPD and PPA will help in developing and implementing maternity care protocols and prevention strategies to reduce such practices and prevent their recurrence.

There is no clear delineation of obstetric maltreatment, both in clinical and community settings. Different terms are used in the literature, including “obstetric violence,” [24] “dehumanized care” [25], “disrespect and abuse” [26], and “mistreatment of women” [27]. We found that 41.5% of the participants reported that they had experienced at least one form of obstetric maltreatment, which was defined as one of seven forms of abuse and neglect. This figure is less than the estimates from the global prevalence where obstetric violence was reported to be approximately 59%, with variations across countries, depending on definitions and measurement tools used [28]. In the West Bank, Palestine, a recent study reported an obstetric mistreatment prevalence of 97.8% [29], where participants reported at least one of six types of mistreatments that were measured via different tools than those used in our study. The striking difference in prevalence can be attributed to the different scales used and to the perception of what constitutes obstetric maltreatment.

Notably, a large proportion of the participants in this study were unfamiliar with the concept of obstetric maltreatment, indicating a normalization of disrespectful practices and a broader lack of awareness around patient rights. This echoes findings from global studies indicating that women often internalize mistreatment during childbirth as routine or even necessary. In low-resource and culturally conservative settings, such behaviour may be viewed as a justifiable effective means of motivating mothers to push during the second stage of labour and ensuring safe delivery [30, 31].

Multiple studies have linked higher rates of mistreatment to deliveries in public hospitals, younger maternal age, low educational attainment, poverty, prolonged or complicated labour, and episiotomy use [28, 32]. These risk factors reflect underlying socioeconomic inequities that affect care quality. In Gaza, these disparities are exacerbated by chronic instability, including the ongoing Israeli blockade, internal displacement, destruction of health facilities and occupation, which severely limit resources and infrastructure. The health system suffers from chronic shortages of staff, equipment, and facilities, making it difficult for providers to deliver respectful, satisfactory high-quality care under extreme pressure [33, 34].

One of the most reported forms of maltreatment in this study was the lack of communication and informed consent. Many women underwent procedures such as vaginal examinations or episiotomies without proper explanation, amplifying their fear, confusion, and helplessness. Inadequate pain relief—despite being a basic patient right [35]—has also been frequently reported.

Although pain threshold among women is highly variable and cannot be accurately measured using an online self-reported questionnaire, having a high number of women (*n* = 458, 64%) reported receiving inadequate analgesics as the commonest form of obstetrics maltreatment warrants implementation of multidimensional pain measurement tools, revising pain management protocols and ensuring its implementation in maternity departments.

These findings reflect deep systemic dysfunction, where overburdened providers deliver hurried care without establishing trust, self-introducing or offering basic patient-centred practices. This results in the erosion of trust and weak rapport and contributes to psychological trauma during what should be a transformative, happy life event [36].

Additionally, the widespread denial of labour companionship is deeply concerning. Numerous studies have confirmed that the benefits of support from a birth companion reduce anxiety, pain, and psychological trauma [10, 35, 37, 38]. However, longstanding policies in Palestinian hospitals often prohibit companions in the delivery room. This practice contributes to negative birth experiences and heightened risks of PPD and PPA, as this study demonstrates.

These factors are strongly associated with psychological distress after childbirth. Our findings align with global evidence linking obstetric maltreatment to increased incidence rates of PPD and PPA [30, 39, 40]. A particularly important result was the significant association between mode of delivery and postpartum mental health. Women who underwent IVDs or CSs were more likely to develop PPDs and PPAs. While these results are consistent with those of some studies, other international studies have reported no such link [41–43]. These differences may stem from varying standards of care. In high-income countries, these procedures are often accompanied by greater support and better postnatal care, potentially reducing psychological harm [44].

Conversely, awareness of maternal rights and recognition of obstetric maltreatment served as protective factors. Women who were knowledgeable about their rights and able to identify mistreatment were significantly less likely to report symptoms of PPD or PPA. This finding underscores the value of health education and empowerment in mitigating trauma. In humanitarian and conflict-affected settings such as Gaza, educational interventions may offer a low-cost, high-impact solution to improve maternal mental health.

Efforts to reduce obstetric maltreatment should include raising awareness of women’s rights during childbirth, making the labour process more supportive, and improving the physical and organizational conditions of maternity facilities. There is a pressing need to train healthcare providers on the principles of respectful and woman-centered care. Second, health systems must institutionalize informed consent processes and ensure that women are adequately prepared for childbirth. Third, psychosocial support services, including screening and referral pathways for PPD and PPA, should be integrated into routine maternity care. Without targeted reform, the normalization of mistreatment perpetuates a harmful cycle of silence and disempowerment that inhibits redress and systemic accountability.

Several limitations should be acknowledged. First, the use of self-reported data may introduce recall or social desirability bias. Additionally, not all women were able to access the internet at the time of data collection to complete the form. The participants were among those who gave birth in the last 5 years, which increases the risk of recall bias. Notably, our subgroup analysis of women who delivered within 12 months of the survey showed findings similar to those of the entire group, suggesting that this was unlikely to be a major source of bias in the study. Second, the cross-sectional design of the study limits causal inference; longitudinal research would be valuable in confirming these relationships over time. Furthermore, reliance on self-reported maltreatment could lead to under- or over-estimation, and ideally, all data should be verified using alternate sources. The rates of reported maltreatment are consistent with what has been reported in other settings. Third, although the study provides rich, context-specific data, healthcare providers are accustomed to working with limited resources and challenging conditions, particularly during times of conflict, as reflected by the high rates of burnout among them [45, 46]. Nonetheless, the findings provide crucial evidence for both local health system reform and broader global health discussions on maternal rights and mental health.

## Conclusion

The study underscores the high prevalence of obstetric maltreatment in the Gaza Strip and establishes a significant association between these experiences and increased risks of postpartum depression and anxiety. These findings highlight the urgent need to integrate respectful maternity care principles into policy and practice, particularly in conflict-affected and resource-deprived settings. When the health system rebuilds after the conflict, it is essential to incorporate principles of respectful maternity care and postpartum support for women who require emergency procedures in labour. Interventions focused on provider training, informed consent, labour companionship, and maternal education are essential to ensure that childbirth is not only safe but also dignified and psychologically supportive.

## Data Availability

The data that support the findings of this study are available from the corresponding author upon reasonable request.

## Abbreviations

LMICs: Low- and middle-income countries
PPD: Postpartum depression
PPA: Postpartum anxiety
WHO: World Health Organization
